# Pre-diagnosing and managing patients with GM1 gangliosidosis and related disorders by the evaluation of GM1 ganglioside content

**DOI:** 10.1038/s41598-019-53995-5

**Published:** 2019-11-27

**Authors:** Rodolfo Tonin, Anna Caciotti, Elena Procopio, Rita Fischetto, Federica Deodato, Maria Margherita Mancardi, Maja Di Rocco, Anna Ardissone, Alessandro Salviati, Antonio Marangi, Pietro Strisciuglio, Giusi Mangone, Arianna Casini, Silvia Ricci, Agata Fiumara, Rossella Parini, Francesco Saverio Pavone, Renzo Guerrini, Martino Calamai, Amelia Morrone

**Affiliations:** 1 Molecular and Cell Biology Laboratory of Neurometabolic Diseases, Neuroscience Department, Meyer Children’s Hospital, Florence, Italy; 2 Metabolic Unit, Meyer Children’s Hospital, Florence, Italy; 3 Divisione Malattie Metaboliche-Genetica Medica, Ospedale Regionale Pediatrico Giovanni XXIII, Bari, Italy; 4 Division of Metabolism, Bambino Gesù Children’s Hospital, IRCCS, Rome, Italy; 5 Unit of Rare Diseases, IRCCS Istituto Giannina Gaslini, Genova, Italy; 6 Divisione Neuropsichiatria Infantile, Fondazione IRCCS Istituto Nazionale Neurologico C. Besta, Milan, Italy; 7 Department of Biotechnology, University of Verona, Verona, Italy; 8 Neurology Unit, Hospital of Vicenza, Vicenza, Italy; 9 Department of Translational Medical Sciences, Section of Pediatrics, Federico II University of Naples, Naples, Italy; 10 Division of Immunology, Section of Pediatrics, Department of Health Sciences, University of Florence and Meyer Children’s Hospital, Florence, Italy; 11 Malattie Metaboliche e Sindromi Malformative Congenite, P.O. Gaspare Rodolico, Catania, Italy; 12 UOS Malattie Metaboliche Rare, Clinica Pediatrica, Ospedale San Gerardo, Monza, Italy; 13 European Laboratory for Non-linear Spectroscopy (LENS), University of Florence, Florence, Italy; 14 Dipartimento di Neuroscienze, Psicologia, Area del Farmaco e Salute del Bambino, University of Florence, Florence, Italy; 15 National Institute of Optics, National Research Council of Italy (CNR), Florence, Italy

**Keywords:** Assay systems, Diagnostic markers, Neurodegeneration, Diagnostic markers

## Abstract

GM1 ganglioside, a monosialic glycosphingolipid and a crucial component of plasma membranes, accumulates in lysosomal storage disorders, primarily in GM1 gangliosidosis. The development of biomarkers for simplifying diagnosis, monitoring disease progression and evaluating drug therapies is an important objective in research into neurodegenerative lysosomal disorders. With this in mind, we established fluorescent imaging and flow-cytometric methods to track changes in GM1 ganglioside levels in patients with GM1 gangliosidosis and in control cells. We also evaluated GM1 ganglioside content in patients’ cells treated with the commercially available Miglustat, a substrate inhibitor potentially suitable for the treatment of late-onset GM1 gangliosidosis. The flow-cytometric method proved to be sensitive, unbiased, and rapid in determining variations in GM1 ganglioside content in human lymphocytes derived from small amounts of fresh blood. We detected a strong correlation between GM1 ganglioside content and the clinical severity of GM1 gangliosidosis. We confirm the ability of Miglustat to act as a substrate reduction agent in the patients’ treated cells. As well as being suitable for diagnosing and managing patients with GM1 gangliosidosis this method could be useful in the diagnosis and management of other lysosomal diseases, such as galactosialidosis, Type C Niemann-Pick, and any other disease with pathologic variations of GM1 ganglioside.

## Introduction

GM1 gangliosidosis is a lysosomal storage disorder mainly caused by the systemic accumulation of GM1 ganglioside due to a deficiency of the beta galactosidase (GLB1) hydrolase. The pathogenic pathway by which GM1 storage leads to cell death involves both the accumulation of toxic products causing inflammatory response and of aberrant mitochondria^1^, a pathogenic pathway common to many neurodegenerative diseases^1^. Also, misregulation of GM1 content is directly involved in Huntington’s and Parkinson’s diseases, in cancer stem cells and in a cancer model in mice^2^.

GM1 gangliosidosis is classified into four groups: infantile, late-infantile, juvenile and adult, based on age of onset, the degree of hepatosplenomegaly, the presence or absence of coarse facies and bone involvement and, above all, the age of onset and degree of neurological involvement which can range from early and severe to slow and progressive^3,4^.

It has recently been reported that Miglustat (butyl-deoxynojirimycin, NB-DNJ) may slow disease progression in patients with juvenile and adult GM1-gangliosidosis by acting as a substrate inhibitor compound^5^. Currently, a number of therapeutic possibilities are being investigated, including glycomimetic small molecules, enzyme replacement therapy and gene therapy^6–10^. Among small molecules acting as substrate reduction agents or chemical chaperons, promising results were reported about the use of N-butyldeoxygalactonojirimycin (NB-DGJ), NB-DNJ and N-octyl 4-epi-β-valienamine (NOEV) in mouse models^11–14^. Studies on AAV9 gene delivery in brain mice models also showed improved nervous system function, increased by systemic AAV9 gene transfer^15,16^. However, therapy is currently limited to supportive care.

Identifying potential biomarkers to gauge the progression of GM1 gangliosidosis would allow the monitoring of existing and new drug therapies^8^. It has been demonstrated that routine serum biomarkers in cats treated with gene therapy can be used to test response to AAV- mediated gene therapy^8^. It has also been shown that inflammatory biomarkers are able to monitor disease progression in GM1 gangliosidosis patient serum and CSF samples^17^. In principle, the liquid chromatography-tandem mass spectrometry (LC-MS/MS) method could be used to directly measure GM1 ganglioside content in dry blood spots^18^. This method, however, requires a specialised LC-MS/MS facility, and a low-throughput sample preparation^18^. More recently, a computer-based imaging method has been used to assess GM1 ganglioside content in cultured fibroblasts^19^.

With the aim of improving diagnostic procedures, we set up a straightforward flow cytometry method able to reveal changes in GM1 ganglioside content in different subtypes of GM1 gangliosidosis patients’ cells. The approach is based on the use of cholera toxin, which, although capable of recognizing several glycans^20^, displays a very high binding affinity for GM1^21–23^. We also evaluated the effect of Miglustat in GM1 gangliosidosis patient cell lines.

The methods here proposed would be suitable in all the lysosomal neurodegenerative diseases in which significant variations of GM1 ganglioside levels occur.

## Methods

### Patients and ethical statements

All patients were diagnosed at a clinical, biochemical and molecular level. Their details are presented in Table 1. The study included 12 samples of lymphocytes from fresh blood and 5 fibroblast lines from patients with all the forms of GM1 gangliosidosis. Heterozygous carrier values of patients’ parents are detailed in Table 2. Three patients had been previously described (Pt4 and 13 in^4^, and Pt17 in^24^).

**Table 1:** Details of GM1 gangliosidosis patients’ cell lines

Fibroblasts	Pt	Ph	Nucleotide changes	Amino acid changes	Mean Florenscence intensity (as from Fig. 1)	% of decrease^‡^ (Miglustat)	Molecular reference
	1.	I	c.176 G > A/c.176 G > A	p.Arg59His/p.Arg59His	132	18.30	13
	2.	I	c.275 G > A/c.1051 C > T	p. Trp92*/p.R351X	129	20.56	4/14
	3.	I	c.1445 G > A/c.1480–2 A >	p.Arg482H/splicing defect	160	29.45	15/16
	4.	J	c.152 T > A/c.602 G > A	p.Ile51Asn/p. Arg201His	121	20	4/17
	5.	J	c.602 G > A/c.247dup1	p.Arg201His/p.Tyr83LeufsX8	117	25	17/this report
**Lymphocytes**	**Pt**	**Ph**	**Nucleotide changes**	**Amino acid changes**	**Median Ratio**^**†**^ **(P/C, as from** **Fig**. **3****)**	**% of decrease**^**‡**^ **(Miglustat)**	**Molecular reference**
	6.	I	c.176 G > A/c.176 G>A	p.Arg59His/p.Arg59His	15.3	n.a	13
	7.	I	c.955 + 2 T > G/c.1480–1 G > A	Splicing defect/splicing defect	9.47	23	this report
	8.	I	c.841 C > T/c.841 C > T	p.His281Tyr/p.His281Tyr	13.5	15	18
	9.	LI	c.572 G > A c.1445 G > A	p.Ser191Asn/p.Arg482His	3.7	n.a	4/16
	10.	J	c.602 G > A/c.1736G > A	p.Arg201His/p.Gly579Asp	1.39	35	15/18
	11.	J	c.176 G > A/c.1313 G > A	p.Arg59His/p.Gly438Glu	1.9	23	13/19
	12.	J	c.602 G > A/c.841 C > T	p.Arg201His/p.His281Tyr	1.95	n.a	17/20
	13.	J	c.1313 G > A/c.1313 G > A	p.Gly438Glu/p.Gly438Glu	1.54	47	19
	14.	J	c.572 G > A/c.1471 G > T	Ser191Asn/Asp491Thr	1.45	5	18/21
	15.*	J	c.602 G > A/c.809 A > C	p.Arg201His/Tyr270Ser	1.23	25	17/this report
	16.*	J	c.602 G > A/c.809 A > C	p.Arg201His/Tyr270Ser	1.29	30	17/this report
	17.	A	c.1068 + 1 G > T/c.1325 G > A	Splicing defect/p.Arg442Gln	1.44	n.a	21/22

Legend. Pt = patient; Ph = phenotype (I = infantile; LI = late infantile; J = juvenile; A = adult); n.a. = not assessed. + Patient in treatment with Miglustat (from 200 mg TID for adults to 100 mg SID in babies with BSA m^2^ < 0.47) at the moment of the blood drawing. The patients reported in bold were previously reported; ^‡^The percentage of GM1 ganglioside decrease after the addition of Miglustat (1 mM) in the medium of cell cultures is calculated from the mean fluorescence values in the case of fibroblasts, and from median values for lymphocytes. ^†^The ratio is calculated between the median value of the GM1 fluorescence intensity of the patient sample and a control labeled and analyzed during the same FACS session. * Pt10 and 11 are brothers.

**Table 2:** Median Ratio^†^ (H/C) of GM1 fluorescence in lymphocytes from heterozygous GM1 gangliosidosis carriers.

Pt	Pt Ph	Nucleotide changes	Amino acid changes	(H/C) Father	(H/C) Mother	(H/C) Son
9	LI	c.1445 G > A	p.Arg482His	3.89		—
		c.572 G > A	p.Ser191Asn		2.52	
12	J	c.602 G > A	p.Arg201His	—	1.66	—
14	J	c.1471 G > T	Asp491Thr		1.23	
17	A	c.1325 G > A	p.Arg442Gln	—	—	1.45

Legend. Pt = patient; Ph = phenotype (LI = late infantile; J = juvenile; A = adult); - = not assessed; H = heterozygous GM1 gangliosidosis carrier; C = unaffected control. ^†^The ratio is calculated between the median value of the GM1 fluorescence intensity of the patient sample and a control labeled and analyzed during the same FACS session.

The institutional committee approving the experiments is Meyer Hospital, Florence, Italy. All experiments were performed in accordance with relevant guidelines and regulations. In keeping with ethical guidelines, all blood and cell samples were obtained for storage and analysis only after the patients’ (and/or their family members’) written informed consent had been obtained, using a form approved by the local Ethics Committee (Ethics Committee of the Meyer Hospital, Florence, Italy). The samples were anonymised and used only for research purposes.

### Cell culture enzyme assays and molecular analyses

Human skin fibroblasts from patients were cultured in Dulbecco’s modified Eagles with fetal bovine serum (10%) and antibiotics. Primary cultures of T- lymphocytes were cultured as previously reported^25^. GLB1 enzyme activity was measured on leukocytes/fibroblasts using the previously reported fluorogenic method^4^.

The amplification and sequencing analysis of genomic fragments was performed as previously reported^4^. The new *GLB1* variants identified in the patients are described according to guidelines of the Human Genomic Variation Society (HGVS) (http://www.hgvs.org/mutnomen/) and using the NM_000404.4 and NP_000395 reference sequences (http://www.ncbi.nlm.nih.gov/gene/).

### *In silico* analysis

The Human Gene Mutation Database (HGMD) (http://www.biobase-international.com/product/hgmd), the 1000 Genomes project database (http://www.1000genomes.org/), including all human genetic variants from the dbSNP short genetic variations database (https://www.ncbi.nlm.nih.gov/snp/) and the Genome Aggregation Database (gnomAD Browser; https://gnomad.broadinstitute.org/), were used to evaluate the polymorphic status of the newly identified amino acid change (p.Tyr270Ser). Single amino acid substitutions were analysed by SIFT (http://sift.jcvi.org/www/SIFT_aligned_seqs_submit.html) PolyPhen (http://genetics.bwh.harvard.edu/pph/) and MutPred (http://mutpred.mutdb.org/) software.

### Cell imaging

Cell imaging was performed on a Nikon Eclipse TE300 C2 LSCM (Nikon, Japan) equipped with a Nikon 60x immersion oil objective (Apo Plan, NA 1.4), with Melles Griot (Argon 488 nm) and Coherent (Sapphire 561 nm) lasers. Emission filters for imaging were 514/30 nm and 595/60 nm. Cells were fixed with 4% PFA, rinsed with PBS (+MgCl_2_ 0,5 mM, + CaCl_2_ 0,8 mM) and permeabilized with 0,075% Triton X. After rinsing with PBS and blocking with 4% BSA PBS, cells were incubated for 20 min with 10 µg/ml biotinylated CTXb, washed and finally labeled with streptavidin Alexa_488 (ThermoFisher, USA, diluted at 1:500) diluted in PBS with 4% BSA. After rinsing again with PBS and water, coverslips were mounted on a glass slide and imaged with LSCM. For LysoTracker™ Red DND-99 (Thermofisher, USA), living cells were grown on 18 mm coverslips and stained following the commercial protocol. Cells were subsequently fixed, permeabilized and labeled with biotinylated CTXb and streptavidin Alexa 488 as described above. For surface labelling, living cells were grown on 18 mm coverslips, rinsed with PBS and incubated with 10 µg/ml biotinylated CTXb diluted in PBS with 4% BSA for 30 minutes on ice, to inhibit endocytic events. After rinsing with PBS, cells were incubated with streptavidin Alexa 488 (1:500) diluted in PBS with 4% BSA for 15 minutes, washed and fixed.

### Flow cytometric analysis

Venous blood samples from GM1 gangliosidosis patients and healthy controls were collected in anti-coagulated tubes with ethylene-diamine tetraacetic acid (EDTA) and processed within 24 h. Peripheral blood mononuclear cells (PBMCs) were obtained by density gradient centrifugation using standard procedures. PBMCs were harvested and washed with phosphate-buffered saline (PBS). BD Cytofix/CytopermTM Fixation/Permeabilization kit (BD Bioscences) was used for fixation and permeabilization according the manufacturer’s instructions. Cells were then incubated with 10 µg/ml biotinylated CTXb diluted in PBS plus 4% bovine serum albumin (BSA) for 30 minutes at room temperature. After washing, the staining was carried out with streptavidin Alexa_488 (1:500) diluted in PBS with 4%BSA for 30 minutes at room temperature. Samples were analyzed on a BD FACS Canto II Flow Cytometer using FACSDIVA software. Lymphocytes were identified by side scattered (SSC) and forward scattered (FSC) light. The positive labelling of GM1 was quantified by the median fluorescence intensity (MFI) of Alexa 488. Small differences in concentration or time of incubation with CTXb - streptavidin Alexa_488, differences in CTXb batch, changes in flow cytometer parameters, or fluctuations in laser intensity, can generate differences in the fluorescence distribution read out. It is therefore essential to run a control treated and analyzed under the same experimental conditions in parallel with the potential carrier/patient. The change in GM1 levels was therefore assessed as the ratio between the MFI of patients and the MFI of healthy age-matched controls analyzed during the same experimental session.

### Statistics

**Figure 1:**
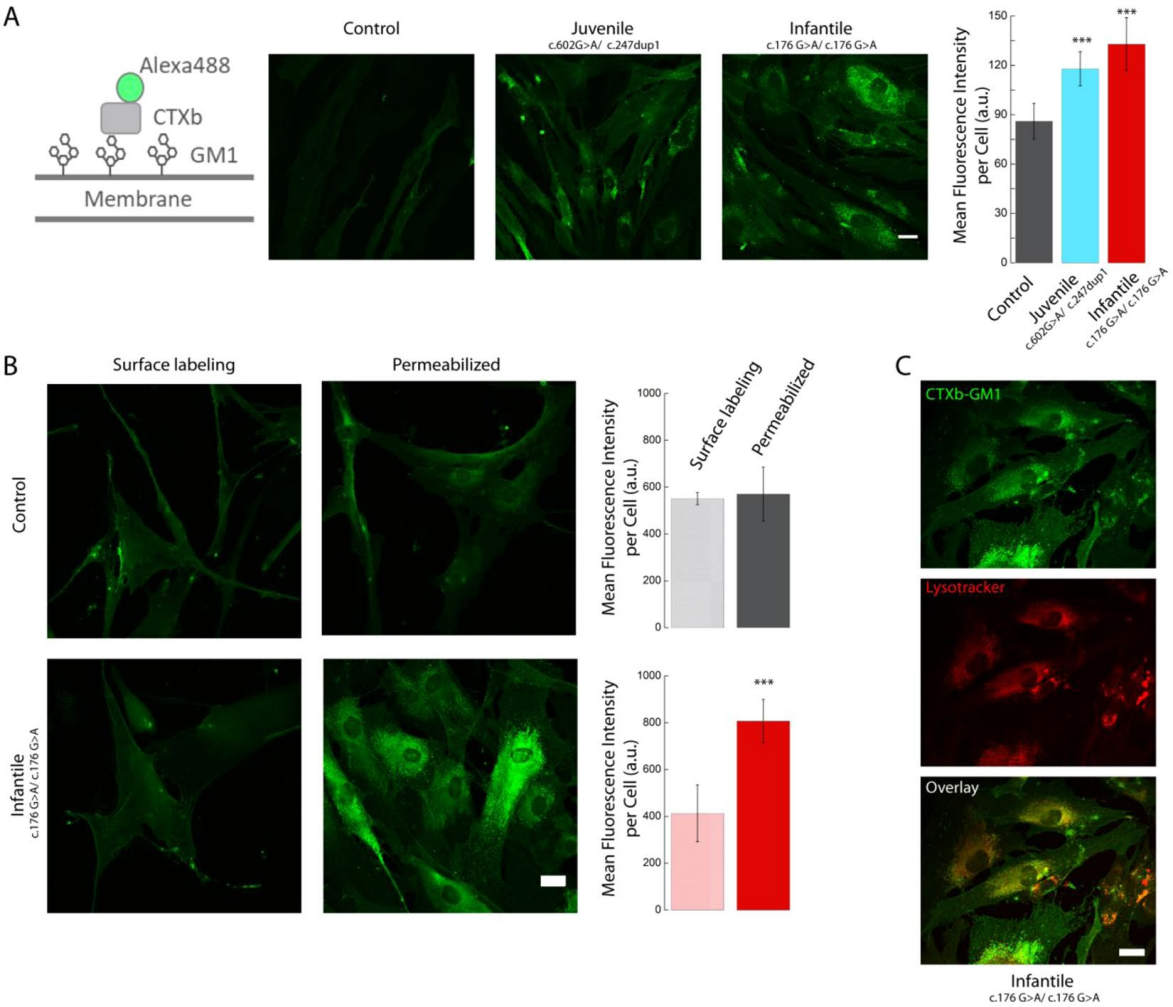
CTXb labelling highlights significant differences in fibroblasts from GM1 gangliosidosis patients, with lysosomal accumulation of GM1 ganglioside. (**A**) Primary cultures of fibroblasts isolated from control and GM1 gangliosidosis patients were fixed, permeabilized and labelled with biotinylated CTXb - streptavidin Alexa_488. Both juvenile and infantile patients show higher values of biotinylated CTXb - streptavidin Alexa_488 fluorescence intensity compared to age-matched control, indicating a related increase in GM1 content. (**B**) Primary cultures of fibroblasts isolated from control and GM1 gangliosidosis infantile patient were imaged either after surface labelling with biotinylated CTXb - streptavidin Alexa_488 and fixation, or after fixation, permeabilization and labelling. The accumulation of GM1 in the infantile patient is predominantly intracellular. (**C**) Primary cultures of fibroblasts isolated from infantile the gangliosidosis patient were incubated with lysotracker red, a lysosomal marker, and subsequently fixed, permeabilized and labelled with biotinylated CTXb - streptavidin Alexa_488. The co-labelling demonstrates that GM1 builds-up at the level of lysosomes. Scale bars 20 µm. >20 cells were analysed for each condition. Error bar S.D. Student’s t test ***P < 0.001.

**Figure 2:**
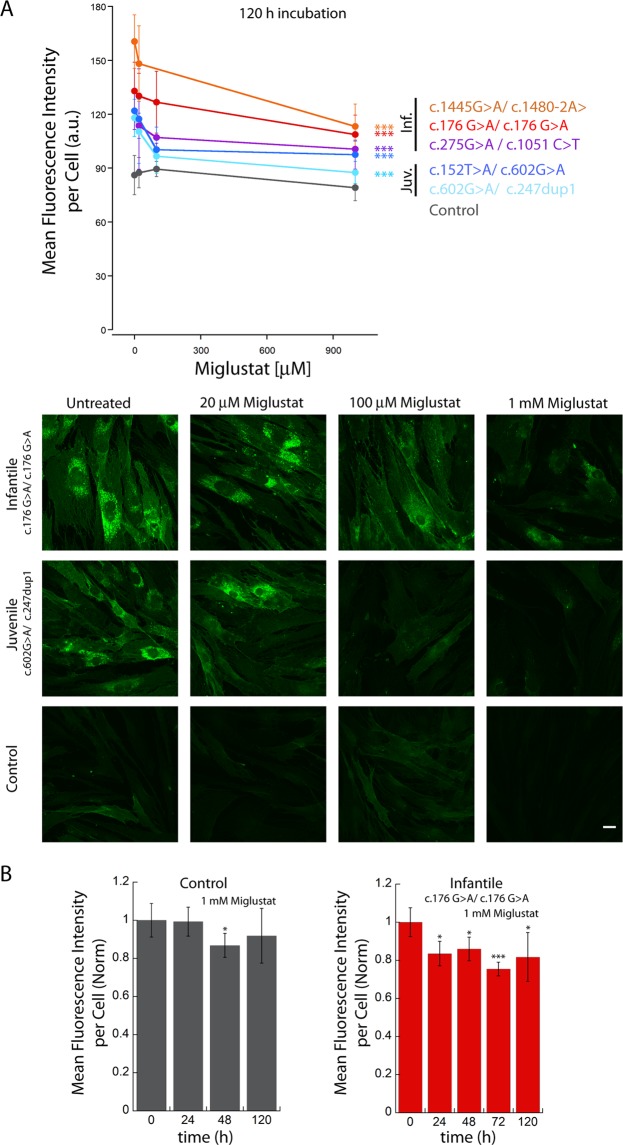
Miglustat reduces the amount of GM1 ganglioside in fibroblasts from GM1 gangliosidosis patients. (**A**) Primary cultures of fibroblasts isolated from patients with different degrees of disease severity were incubated for 5 days with different concentrations of Miglustat and subsequently fixed, permeabilized and labelled with biotinylated CTXb - streptavidin Alexa_488. The mean fluorescence intensity per cell decreases with increasing concentrations of Miglustat, both in infantile and juvenile patients. At the bottom, representative images of the decrease for control, juvenile and infantile patients. Scale bar 20 µm > 20 cells were analysed. Error bar S.D. ANOVA test ***P < 0.001. (**B**) Primary cultures of fibroblasts isolated from control and infantile patient were incubated for different lengths of time with 1 mM Miglustat and subsequently fixed, permeabilized and labelled with biotinylated CTXb - streptavidin Alexa_488 > 20 cells were analysed. Error bar S.D. Student’s t test *P < 0.05, ***P < 0.001.

**Figure 3:**
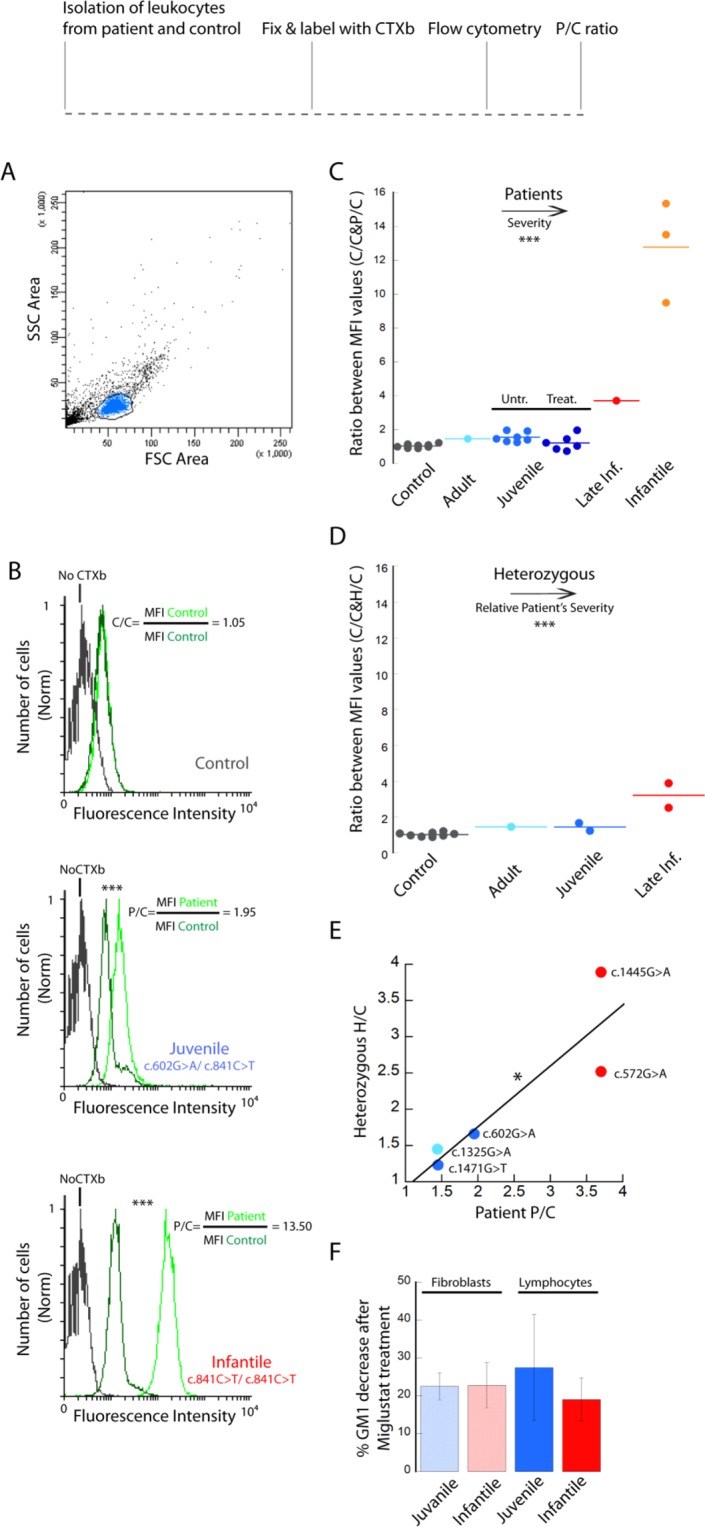
CTXb labelling of GM1 ganglioside and FACS analysis of lymphocytes from the fresh blood of patients with different degrees of GM1 gangliosidosis severity. Lymphocytes were fixed and labelled with biotinylated CTXb - streptavidin Alexa_488. (**A**) Example of control cells analysed with flow cytometry. The SSC vs FSC PBMCs plot is gated to identify the lymphocytes population (in blue). (**B**) Examples of CTXb - streptavidin Alexa_488 fluorescence distribution. Cells within the lymphocyte gate defined in (**A**) are represented in histograms to evaluate the relative amount of GM1 labeled with biotinylated CTXb-streptavidin Alexa_488 in patient and healthy control. The fluorescence distribution of patient lymphocytes (light green peak) shows a marked increase with respect to age-matched control cells (dark green peak) stained in the same experimental session. MFI, median fluorescence intensity. Unstained cells (grey peak) display low levels of auto-fluorescence. Wilcoxon test ***P < 0.001. P/C (or C/C) ratio values are obtained dividing the MFI of the patient (or additional control) distribution by that of the control. (**C**) P/C values increase with increasing pathological severity of the patients. Juvenile patients undergoing Miglustat treatment show P/C values comparable to those obtained from age- matched control samples. ANOVA test ***P < 0.001. (**D**) H/C ratio values, obtained dividing the MFI of the heterozygous distribution by that of the control values, increase with increasing pathological severity of the affected relative. ANOVA test ***P < 0.001. (**E**) A significant linear correlation (R = 0.89, *P < 0.05) is found between the P/C and the H/C values of patient and heterozygous relatives. (**F**) Cultured lymphocytes isolated from juvenile and infantile patients treated with 1 mM Miglustat for 5 days and analyzed with FACS show a decrease in median GM1 ganglioside content analogous to that measured with confocal microscopy in juvenile and infantile patient fibroblasts incubated under the same conditions (see Fig. 2). Error bar S.D. > 5000 cells were analysed for each condition.

In Fig. 1, data are expressed as mean ± Standard Deviation (S.D.) and statistical significance evaluated using Student’s t test. In Fig. 2A, data are normalized to the value at time 0 and expressed as mean ± Standard Deviation (S.D., calculated in this case as absolute error from independent measurements) and statistical significance evaluated using Student’s t test. In Fig. 2B, data are expressed as mean ± S.D. and statistical significance evaluated using one-way ANOVA. The t-test was used to compare two data sets while one-way ANOVA was used to evaluate the influence of one factor (concentration of drug, or severity of the pathological condition) in multiple comparisons. In Fig. 3B, statistical significance was evaluated using the nonparametric Wilcoxon test for distributions that are not normally distributed. In Fig. 3C,D statistical significance was evaluated using one-way ANOVA. In Fig. 3E, statistical significance was evaluated with a correlation test. Statistical analysis was performed using the software KaleidaGraph, or resource available on-line (https://www.graphpad.com/quickcalcs/PValue1.cfm). In each figure legend, we report the statistical tests employed. The minimum number of cells analysed is indicated in the respective legends.

## Results

### Molecular analysis

*GLB1* molecular analysis of the patients is reported in Table 1. Parental carrier status was ascertained in all cases. As shown in Table 1, we found five new heterozygous mutations in the *GLB1* gene in five different patients. Of these, three mutations are nucleotide changes resulting in splicing defects (c.955 + 2 T > G, c.1480–1 G > A and c.1068 + 1 G > T), one is a single nucleotide duplication (c.247dup1) and one is a missense mutation [c.809 A > C (p.Tyr270Ser)]. The p.Tyr270Ser amino acid change was found in combination with the p.Arg201His mutation, in Pt16 and Pt17. The p.Tyr270Ser amino acid is located near to the catalytic pocket that includes the residues Glu188-Glu268^26^. A different mutation (p.Tyr270Asp), involving the same amino acid, is predicted to interact with the ligand binding pocket^27^. The p.Tyr270His mutation is predicted to cause changes in the catalytic site at Glu268 or to alter the ordered interface or transmembrane domains (MutPred software). All *in silico* prediction tools suggested that such a mutation would be severe, confirming the above-mentioned data from the literature. Conversely, the p.Arg201H mutation is a mild mutation reported in GM1 gangliosidosis type II and III patients^4,28,29^. Thus, the phenotype of Pt16 and Pt17, who present a juvenile form of the disease, derives from the partial residual GLB1 activity of the mutated protein carrying the p.Arg201His mutation.

### Imaging of GM1 accumulation in lysosomes in patients affected by GM1 gangliosidosis

Cultured human fibroblasts from control and from juvenile (c.602G > A/c.247dup1) (p.Arg201His/ p.Tyr83LeufsX8) and infantile (c.176G > A/c.176G > A) (p.Arg59His/p.Arg59His) patients with GM1 gangliosidosis were fixed, permeabilized and labelled with the biotinylated b subunit of cholera toxin (CTXb), known for its very high affinity with GM1 ganglioside, and streptavidin Alexa_488 fluorophore (Fig. 1). As predicted, images of juvenile and patient fibroblasts acquired with a confocal laser scanning microscope (CLSM) displayed, respectively, 37% and 55% increases in mean fluorescence intensity per cell, compared to fibroblasts derived from an unaffected donor (Fig. 1A).

In order to evaluate if GM1 increased exclusively in intracellular organelles or also at the level of the plasma membrane, we compared the amount of GM1 in fibroblasts that had either undergone fixation, permeabilization and labelling, or solely surface labelling and fixation, in the case of an infantile patient (c.176G > A/c.176G > A) (p.Arg59His/p.Arg59His) and of an unaffected donor (Fig. 1B). As the amount of GM1 ganglioside present on the plasma membrane was comparable in patient and control fibroblasts, it is possible to conclude that the dramatically higher levels of GM1 ganglioside observed in permeabilized pathological samples are attributable to larger intracellular build-ups.

In agreement with GM1 gangliosidosis models, when infantile patients’ (c.176G > A/c.176G > A) (p.Arg59His/p.Arg59His) fibroblasts were co-labelled with CTXb and lysotracker, a specific lysosomal marker, a high degree of co-localization was found between the intracellular deposits of GM1 ganglioside and the lysosomal compartments (Fig. 1C).

Our results indicate that CTXb - streptavidin Alexa_488 can be used to measure the accumulation of GM1 ganglioside in lysosomes in fibroblasts from GM1 gangliosidosis patients, and that the degree of GM1 labelling is proportional to the degree of disease severity.

### Treatment with Miglustat reduces the accumulation of GM1 ganglioside in fibroblasts

We treated patients’ fibroblasts with the substrate inhibitor Miglustat, which we predicted would prevent the synthesis of new GM1 ganglioside. Primary cultures of fibroblasts from patients characterized by different degrees of severity (juvenile and infantile) were incubated for 120 hours with different concentrations of Miglustat, ranging from 20 µM to 1 mM (Fig. 2A). Cells were fixed, permeabilized and labelled with CTXb at the end of the treatment, and subsequently imaged with CLSM. Also in this case the amount of labelling in untreated fibroblasts from different patients was correlated to the degree of severity (Table 1). In all cases, even doses of Miglustat as low as 20 µM induced a decrease of 5–15% in total GM1 ganglioside content. The maximum decrease was observed at 1 mM Miglustat and was around 20–30%.

Primary cultures of fibroblasts from an infantile patient (c.176G > A/c.176G > A) (p.Arg59His/p.Arg59His) and from an unaffected donor were incubated for different times with 1 mM Miglustat (Fig. 2B). While only a modest decrease in CTXb - streptavidin Alexa_488 fluorescence was observed in the fibroblasts of the unaffected donor after 48 hours, those of the infantile patient already displayed a significant reduction after 24 hours incubation, which did not then decrease substantially after 120 hours.

### Flow cytometry analysis of GM1 ganglioside content in lymphocytes

We applied the CTXb - streptavidin Alexa_488 labelling approach to fluorescence flow cytometry, a more rapid, unbiased, and statistically solid method than microscopy imaging, and we quantified the amount of GM1 ganglioside in lymphocytes isolated from human fresh blood samples. As for fibroblasts, lymphocytes were fixed, permeabilized and labelled with CTXb. Lymphocytes were identified by forward and side scatter gating (Fig. 3A). When control lymphocytes were permeabilized and labelled with CTXb coupled to Alexa_488, an increase in median fluorescence intensity (MFI) of approximately 2.5–3 folds was detectable with respect to basal cellular auto-fluorescence (Fig. 3B). MFI values were considered instead of mean values as the fluorescence of the cells does not follow a normal distribution. In order to quantify the changes in fluorescence intensity distribution between controls and patients, we calculated the ratio between MFI values of samples of patients and controls (P/C) that simultaneously underwent the same procedure of fixation and labelling. Ratio values obtained by dividing the MFI of different controls (C/C) analysed in the same experimental session show a range between 0.8 and 1.2 (Fig. 3C, Table 1); the fluorescence values of control samples that were processed during different sessions show a higher variability dependent on small changes in labelling efficiency, making it inappropriate to simply compare absolute MFI values from experiments performed in different days. Therefore, we ran a fresh age-matched control in parallel with the patient’s sample, in order to obtain a proper reference for comparison. Juvenile patients exhibited P/C values between 1.4 and 3.8 (Fig. 3C, Table 1). The P/C values of juvenile patients under treatment with Miglustat were comparable to the control values (C/C). As expected, the P/C values comparing infantile patients and controls reached values as high as 10. It is notable that the P/C value of the only late infantile patient was between juvenile and infantile values, while the adult P/C was comparable to the juvenile values. These results show a strong correlation between the degree of severity of the pathological condition and the P/C values obtained by CTXb labelling.

Interestingly, in heterozygous carrier samples, the heterozygous/control (H/C) MFI ratio varied along with the pathological condition of the family member affected (Table 2, Fig. 3D), and correlated significantly with the gravity of the single point mutation carried (Fig. 3E). For example, the heterozygous carrier with the most severe mutation (c.1445G > A) (p.Arg482His) showed the highest H/C fluorescence ratio.

We also cultured lymphocytes isolated from the blood of juvenile and infantile patients, with or without 1 mM Miglustast for 5 days. Lymphocytes were then labelled and analysed with flow cytometry as above. When the MFI values of treated and untreated cells were compared, a decrease in GM1 ganglioside content of about 20–30% was observed (Fig. 3F), in line with the results obtained in fibroblasts with CLSM (Figs. 2B and 3F). Notably, the decrease in GM1 ganglioside content after 1 mM Miglustat treatment appeared to be constant and not dependent on the severity of the pathology or the cellular type.

## Discussion

In all subtypes of GM1 gangliosidosis neurological symptoms appear because GM1 ganglioside accumulation induces neurodegenerative processes in the nervous systems^30^. A review of the literature confirmed that neurological involvement is invariably present in all GM1 gangliosidosis patients^31^. The cortical and cerebellar damage is severe in the infantile and late-infantile forms of the disease^3,4^. Juvenile patients progressively lose psychomotor milestones and develop dementia, spastic quadriparesis, progressing to vegetative state and death, usually within the third decade of life^3^. They can also experience progressive myoclonus epilepsy^32^ or refractory tonic clonic seizures^30^. Dystonia, parkinsonism and dysarthria are the main neurological signs of the adult form of the disease^33^.

In many patients with GM1 gangliosidosis, whose symptoms manifest in the juvenile period or in adulthood, distinctive features of a lysosomal storage disorders, e.g. cherry red spot, marked coarse facies and hepatosplenomegaly, are often missing, making diagnosis complex^30^. Beside, inherited late onset neurodegeneration is a clinical manifestation of several late-onset lysosomal diseases^34^ and of many neurodegenerative diseases with different etiologies^35^. A sure diagnosis of GM1 gangliosidosis is even more challenging because of its low incidence, currently reported as 1:100.000–1:200.000 live births^3,36^. Three different reports, which describe the employment of exome/whole genome sequencing to identify GM1 gangliosidosis, show that diagnosis is often very delayed. In the patients described diagnosis was not suspected for some years^30,35,37^, in one patient for over a decade^35^, because of nonspecific symptoms and/or brain MRIs. These observations suggest that the disease may be substantially underestimated.

Current diagnostic tools for lysosomal storage diseases are based on enzymatic assays, and/or genetic screening, although glycosphingolipid (GSL) evaluations can be used in the differential diagnosis of sphingolipidoses. Conventional GSL analyses are currently performed by thin- layer chromatography (TLC), recently improved by immunochemical detection and HPLC to overcome the low resolution limits of conventional TLC^18^. It has been proposed that ganglioside profiling could also be performed by using LC-ESI- MS^18^. However, most of these techniques, including LC-ESI-MS, require extensive sample preparation and/or the availability of expensive MS/MS facilities. A simple and cheap method to evaluate GM1 ganglioside storage in peripheral blood samples is not yet available.

We here report a simple, quick and affordable approach to measuring GM1 ganglioside in fixed fibroblasts and in lymphocytes derived from patients with all the GM1 gangliosidosis forms (infantile, late infantile, juvenile and adult). We demonstrated that the substrate inhibitor Miglustat reduces the levels of GM1 ganglioside in cultured cells from GM1 gangliosidosis patients, and that our highly sensitive labelling approach is suitable for monitoring changes in GM1 ganglioside content. Our results demonstrate that fluorescent labelling with CTXb of lymphocytes isolated from fresh blood samples and analysis with flow cytometry is capable of differentiating between patients with early onset and late onset GM1 gangliosidosis. In addition, our method identified heterozygous carriers from samples of age-matched controls confirming that the method is sensitive and accurate.

In principle, GM1 ganglioside could be used as a general biomarker, suitable in clinical assessments or in the management of other neurodegenerative lysosomal diseases. More specifically, the lysosomal storage disorder Galactosialidosis (GS), caused by the loss of function of PPCA/CTSA, results in the secondary combined deficiency of GLB1 and NEU1^38^. GS, currently classified as a sphingolipidosis or a glicoproteinosis, in its features of sphingolipidosis is biochemically characterized by the storage of GM1 ganglioside, due to a deficiency of GLB1, which cleaves the beta-linked galactose of lactosylceramide, asialofetuin, oligosaccharides and, above all, GM1 ganglioside^4^. The storage of GM1 ganglioside in the early endosome compartment of Chinese hamster ovary cells [NPC(-) cells] and in NPC1-/- mouse tissues is documented^39–41^. An improved diagnostic procedure would also benefit patients with NPC^42^. The “filipin test”, performed on cultured fibroblasts, is the historical gold standard used to establish a diagnosis of NPC^42^. However, although a recent algorithm has improved the efficacy of the filipin test, in up to 15% of all referrals, results remain inconclusive unless molecular analysis is performed^42^. Thus, an improved diagnostic procedure and/or the identification of potential biomarkers to follow emerging therapies, would also benefit patients affected by NPC^42,43^.

In addition, a GM1 gangliosidosis variation may be detected in molecular defects involving the biosynthesis of gangliosides. Defects occurring upstream of the GM1 ganglioside biosynthesis, such as the deficit of GM3 synthase and of N-acetyl-beta-glucosaminyl-glycoprotein 4-beta-N-acetylgalactosaminyltransferase 1, responsible for Spastic Paraplegia 26^44^, could cause a significant depletion of GM1 ganglioside levels. The diagnostic methods we here propose could be useful for detecting GM1 gangliosides levels in the lysosomal disorders NPC and GS and in the GSL biosynthesis defects such as the deficit of GM3 synthase and Spastic Paraplegia 26.

We also stress that although preliminarily evaluated in a restricted number of samples (n = 5), the flow cytometric method showed a high sensitivity in detecting unaffected heterozygous GM1 gangliosidosis carriers. Thus, our data suggest that the method is potentially suitable for high-throughput screening programs of this sphingolipidosis.

We envisage that the methodological approach we have used, which is reliable, fast and routinely feasible in laboratories equipped with a fluorescence flow cytometer, could be incorporated into the clinical laboratory practices for large population studies in the near future.
